# Emerging *Mycobacteria* spp. in Cooling Towers

**DOI:** 10.3201/eid1501.071356

**Published:** 2009-01

**Authors:** Isabelle Pagnier, Michèle Merchat, Didier Raoult, Bernard La Scola

**Affiliations:** Unité des Rickettsies, Marseille, France (I. Pagnier, D. Raoult, B. La Scola); Climespace Service Recherche & Développement, Paris, France (M. Merchat)

**Keywords:** Mycobacteria, ameba, cooling tower, letter

**To the Editor:** The importance of nontuberculous mycobacteria (NTM) in various clinical situations recently has increased. Members of the *Mycobacterium avium* complex (MAC) cause a high percentage of infections in persons with acquired immunodeficiency syndrome. Some species are considered emerging pathogens, particularly those of the *M. chelonae–M. abscessus* group. They occur not only in immunocompromised persons but also in persons without predisposing conditions. Although sources of infection are considered to originate from the human environment, until now cooling towers were not clearly demonstrated to be one of these contamination sources, despite being suspected (*1*). Natural streams, groundwater, brook waters, and swamps already were reported to contain different species of NTM. Constructed environments, such as hospital hot water systems, aerosols from showers, ice machines, swimming pools, dental unit water, endoscopes, and bronchoscopes are other sources (*2*). Other studies have shown members of MAC in drinking-water distribution systems (*3*).

A 1999 study in South Africa reported the presence of mycobacteria in cooling towers but reported no details about species (*4*). In 2003, cooling towers were reported to be a potential source of slow-growing mycobacterial species (*1*). Some mycobacterial species recently were shown to survive within amebae (*5*). We aimed to study the population of ameba-associated mycobacterial species in 3 cooling towers using a co-coculture method.

These cooling towers (E, H, and O), located in downtown Paris, France, were sampled in May 2006. Water was taken in a sample point of the cooling circuit located just before the entrance of the tower basins. Because these cooling towers are regularly treated by oxidizing biocide BCDMH (1-bromo-3-chloro-5,5-dimethylhydantoin) to prevent development of *Legionella* spp., no *Legionella* spp. or *Legionella-*like amebal pathogens were isolated.

Two liters of water samples were filtrated through 0.22-µm pore-sized filters that were injected onto amebal microplates as previously described (*6*) and incubated at 32°C. We screened amebal microplates by examination under inverted microscope and by Ziehl-Nielsen, Gram, and Gimenez staining. Positive wells were subcultured on axenic medium and incubated at 32°C for 10 days. Acid-fast isolates were identified by using partial *rpo*B gene amplification and sequencing with Myco-F and Myco-R primers (*7*). These primers did not allow amplification of partial *rpo*B gene for the 6 *M. phocaicum* strains*.* We thus used Myco-F/Myco-Rbon (5′-AGCGGCTGCTGGGTGATCAT-3′) primer pair for *M. phocaicum* (*8*). We compared these sequences with the *rpo*B gene sequence of *Mycobacteria* type strains available in the GeneBank database by using BLASTn on the NCBI website (www.ncbi.nlm.nih.gov).

We observed bacteria growing in amebae. Subculture on axenic media led to polymicrobial cultures and allowed isolation of 33 mycobacterial strains (Table). All these strains were submitted to molecular identification. The 33 isolates corresponded to 5 mycobacterial species: *M. fortuitum, M. conceptionense, M. chelonae,* *M. chimaera,* and *M. phocaicum* (Table). Some of these mycobacteria already had been shown to survive in free-living amebae and to be implicated in human diseases, such as *M. chelonae* (*5*). The same author demonstrated recently that 26 environmental mycobacteria survived in the trophozoites and cysts of *Acanthamoeba polyphaga* (*5*). The recently described species *M. phocaicum* was isolated only in samples from humans and was associated with chronic pneumonia (*9*). However, the natural source of this species is still unknown. *M. fortuitum* was described as an opportunistic *Mycobacterium* associated with disseminated infections in a leukemia patient or in furunculosis after footbath in nail salons. *M. conceptionense* belongs to the *M. fortuitum* group and was isolated in a posttraumatic osteitis inflammation (*10*). *M. chimaera* belongs to MAC and has been isolated only in patients with pulmonary disorders but not from immunocompromised patients. The authors reported that the isolate showed unusually high virulence. We isolated this species for the first time in an environmental sample. It could belong to the transitory flora of the H cooling tower because it was isolated only once in our samples. Cooling towers already had been investigated for slow-growing mycobacteria (*1*), and our study showed they also can be an environmental source of rapidly growing NTM pathogens. In our procedure for amebal co-cultures, incubation cannot last >10 days because amebae do not survive longer in Page’s amebal saline buffer. Moreover, agar plates were incubated for 10 days only, which explains why we could recover only rapidly growing mycobacteria. The cooling towers, already known as a source of dissemination of *Legionella* spp., may disseminate mycobacteria in aerosols and be a previously nonrecognized source of infection.

**Table:** Identification of isolated nontuberculous mycobacteria according to their *rpoB* gene sequence similarity and GenBank accession numbers

Closest officially described species (*rpoB*)	Isolates (no.), N = 33	Range in % gene similarity to type strain (GenBank accession no.)	Accession numbers of sequences of isolates
*Mycobacterium chelonae*	O (2), E (2)	99.7 (AY147163)	EU770577
*M. conceptionense*	O (12)	99.2–99.9 (AY859695)	EU770583, EU770584
*M. fortuitum*	E (2), H (5), O (3)	99.5–99.7 (AY147165)	EU770578, EU770579, EU770580
*M. chimaera*	H (1)	99.7 (EF521908)	EU770576
*M. phocaicum*	E (5), O (1)	98.3–98.6 (AY859693)	EU770581, EU770582
